# Structural Relationship between Korean Adolescent’s Sports Participation, Optimism, Pessimism, Self-Regulation, and Coronavirus-Related Stress in the Pandemic Situation

**DOI:** 10.3390/ijerph182010645

**Published:** 2021-10-11

**Authors:** Ho-Hyun Song, Dae-Jung Lee

**Affiliations:** 1 Department of Physical Education, Korea National University of Education, Chungju-si 28173, Korea; hohyunss@jbedu.kr; 2 Department of Physical Education, Jeonbuk National University, Jeonju-si 54896, Korea

**Keywords:** adolescent, coronavirus-related stress, optimism, pessimism, physical activity, self-regulation

## Abstract

This study aimed to examine the relationships between sports participation, optimism/pessimism, self-regulation, and coronavirus-related stress in Korean adolescents during the pandemic situation. Specifically, we attempted to offer valuable information that could help to alleviate coronavirus-related stress in adolescents by promoting participation in sports and the development of optimism and self-regulation. To achieve this aim, we conducted an online survey of 836 Korean adolescents in the pilot and main studies. Confirmatory factor, frequency, path, reliability, descriptive statistical, and multimedia analyses were performed. Our findings indicated several differences for each variable according to demographic characteristics. Sports participation exerted a positive effect on optimism (*p* < 0.001) and self-regulation (*p* < 0.01) and negative effects on coronavirus-related stress (*p* < 0.05) and pessimism (*p* < 0.001). In addition, optimism exerted a positive effect on self-regulation (*p* < 0.001) and a negative effect on coronavirus-related stress (*p* < 0.001), while pessimism exerted a negative effect on self-regulation (*p* < 0.01) and a positive effect on coronavirus-related stress (*p* < 0.001). Further analysis indicated that self-regulation had a negative effect on coronavirus-related stress (*p* < 0.05). These findings highlight the need for youth educational institutions to encourage adolescents to participate in sports and for organizing bodies to suggest various policies and provide education that can assist them in properly coping with and overcoming coronavirus-related stress by strengthening their optimistic attitude and self-regulation ability.

## 1. Introduction

In 2020, the World Health Organization (WHO) declared a “public health emergency of international concern (PHEIC)” due to the worldwide spread of coronavirus disease-19 (COVID-19), declaring the situation a “pandemic” [1]. Despite government measures to contain COVID-19 outbreaks, more than 198 million confirmed cases and more than 4.2 million deaths have occurred worldwide as of the end of July 2021, and 42,000 new deaths and three million new cases of COVID-19 are being reported each day [2]. In addition, the dramatic changes and threats brought about by the COVID-19 pandemic have had a profound impact on mental as well as physical health. Although many previous clinical studies have been conducted worldwide to develop a vaccine for COVID-19, its impact on mental health and related services has been largely underestimated [3,4]. Given the increasing number of mental health problems associated with physical illness, systematic investigations in these areas are necessary to understand the extent of the issue and apply therapeutic and preventive strategies [5,6].

According to a recent study, over 25% of the population in China has experienced severe symptoms related to anxiety and stress due to unexpected and unusual situations and fears caused by COVID-19 [7]. A study of Korean adults reported that after the COVID-19 outbreak in Korea, 42.8%, 36.1%, and 23.6% of the individuals reported experiences of depression, anxiety, and stress, respectively [8]. Moreover, in the case of the United Kingdom, a study on mental health after the onset of the COVID-19 pandemic showed an increase in mental health problems in 10.3% and 16.4% of men and women, respectively [9]. Adolescents in particular have encountered mental health concerns such as anxiety, depression, and obsessive–compulsive disorder due to the continuous stress imposed by COVID-19 [6,10,11]. Current evidence suggests that these mental health areas are closely related to physical activity, which is known to be effective in maintaining and improving mental health [12]. Indeed, physical activity in adolescents not only affects health treatment and outcomes (e.g., bone density, physical activity in adulthood), but also relieves symptoms of psychological tension such as stress, depression, and anxiety [13]. In other words, the data suggest that there is a significant correlation between sports activity and stress levels in adolescents.

Stress exhibits a well-known association with self-regulation. Self-regulation is a mental and behavioral process that results in conformity with the self-concept and personal goals by putting one’s self-concept into action, modifying one’s behavior, or changing the external environment [14]. In adolescence, the ability to control impulses and carefully plan, solve, and evaluate problems related to oneself becomes necessary. Research has indicated that self-regulation is related to the process of resource depletion [15]. Intentional efforts to increase flexibility and adaptability to the environment by controlling oneself are processes that consume limited resources. Due to the limited nature of self-regulation described in terms of the strength model or ego depletion, those who continue to self-regulate beyond limitations experience fatigue, resulting in a failure to self-regulate [16]. Accordingly, further research is required to identify the variables that prevent failure of self-regulation due to fatigue, such as skill, motivation, fatigue, self-efficacy, emotion, and personality variables [17].

Therefore, when examining the relationship between self-regulation and stress, it is necessary to investigate the control variables that can prevent fatigue of self-regulation, enable self-regulation, and consequently reduce stress. Optimism, which can be considered a moderator variable, is a belief that good things will happen in one’s life [18], and it is a concept that reflects positive and hopeful emotions for the future. Positive emotions have the effect of offsetting negative emotions [19], and positive emotions resulting from an optimistic attitude can attenuate negative emotions and fatigue associated with self-regulation. In fact, studies have reported that optimism positively affects stress and plays a key role in improving self-regulation [20]. In addition, optimism allows self-regulation to continue even in difficult situations [21]. Therefore, optimism is thought to not only affect stress, but also the influence of self-regulation on stress.

As noted above, previous research has demonstrated a positive relationship between participation in physical activity and self-regulation. In addition, participation in physical activity, optimism, and self-regulation exert a negative effect on stress. However, these studies have only examined partial relationships between the factors, such as the relationship between physical activity and self-regulation [22] or that between self-regulation and stress [23]. This is limited in that it does not provide information regarding the comprehensive structural relationships between participation in physical activity, optimism/pessimism, self-regulation, and stress. In addition, very few studies have measured the stress of adolescents arising from the special circumstances of COVID-19. For example, it is difficult to directly participate in physical activities due to pandemic-related measures such as social distancing, restrictions on the use of schools and external sports facilities, conversion of physical education classes to the online format, and restrictions on watching sports in person. Given this situation, the number of studies investigating measures that can help alleviate “COVID-19-related stress” among adolescents remains insufficient.

Therefore, in this study, we empirically investigated the relationships between participation in sports, optimism/pessimism, self-regulation, and coronavirus-related stress among adolescents. Specifically, we aimed to provide practical information that can minimize stress among adolescents in the context of COVID-19. To achieve this goal, the following research hypotheses were established based on previous studies and various theories. First, we hypothesized that there would be differences in sports participation, optimism, pessimism, self-regulation, and coronavirus-related stress according to sex, school level, frequency of physical activity participation per week, amount of time spent engaged in physical activity, and duration of physical activity participation (Hypothesis 1). Second, that sports participation would positively/negatively affect optimism, pessimism, self-regulation, and coronavirus-related stress (Hypothesis 2). Third, that optimism and pessimism would positively/negatively affect self-regulation and coronavirus-related stress (Hypothesis 3). Fourth, that self-regulation would exert a negative effect on coronavirus-related stress (Hypothesis 4).

## 2. Methods

### 2.1. Participants

In this study, 836 adolescents were selected as the participants using the convenience sampling method, which is a non-stochastic sampling method, in the Republic of Korea. Specifically, 210 adolescents were selected for the pilot test from one middle school and one high school in the Jeollabuk-do Province, and 626 adolescents were selected for the main study from one middle school and one high school in the Chungcheongnam-do Province, in the Gyeonggi-do Province, in the Incheon Province, in the Jeollabuk-do Province, and in the Jeollanam-do Province, respectively. Only middle and high school adolescents were included in the study. There were no specific exclusion criteria. The demographic characteristics of the participants are presented in Table 1. To collect data, we conducted a self-reported online survey (presented using Never Forms) from 12 July to 22 July 2021. This study was conducted after obtaining ethical approval from the Institutional Review Board of Jeonju National University of Education (JNUE 21 January 2021).

### 2.2. Instruments

Demographic characteristics of the participants were evaluated using questions concerning sex, school level, frequency of participation in exercise, exercise intensity, and period of participation in exercise. Participation in sports was defined based on the sports participation classification model of Snyder [24], and the reliability and validity of the scale used were verified by Lee et al. [25]. Snyder’s [24] classification of sports participation consisted of three sub-variables (cognitive, behavioral, and affective participation) with three items each, and a total of nine items. Optimism and pessimism were assessed using a scale with verified reliability and validity as reported by Chang et al. [26]. The scale consisted of seven items related to optimism and pessimism each. Self-regulation was assessed by modifying and supplementing the scales used by Gaumer Erickson et al. [27]. Self-regulation ability was assessed using four questions related to reflecting, adjusting, monitoring, and planning. Reflecting involves reflecting on what can be done better in the future. Adjusting involves making adjustments by implementing specific strategies when things do not go as planned. Monitoring involves immediate monitoring of the progress and interventions toward the goals. Planning has to do with strategizing and making clear what is to be achieved. Coronavirus-related stress was assessed using the COVID Stress scale [28]; its reliability and validity have been verified [29].

The COVID Stress scale includes five items related to danger and contamination, four items related to xenophobia, four items related to traumatic stress, and four items related to compulsive behavior. Experience of sports participation, optimism and pessimism, self-regulation, and coronavirus-related stress were rated as follows: *strongly agree* (5 points), *agree* (4 points), *neutral* (3 points), *disagree* (2 points), *strongly disagree* (1 point). A score closer to 5 indicates a higher awareness of the variable, and a score closer to 1 indicates a lower awareness of the variable.

### 2.3. Reliability and Validity of the Instrument

Although the questionnaires used in this study were previously validated, confirmatory factor analysis was performed to verify the validity of the scales as it was possible that the sociocultural and economic backgrounds of the participants of this study differed from those of the previous samples. Confirmatory factor analysis revealed that the fitness of the proposed model was as follows: root-mean-square residual (RMR) = 0.055, normed fit index (NFI) = 0.893, comparative fit index (CFI) = 0.917, incremental fit index (IFI) = 0.918, root-mean-square error of approximation (RMSEA) = 0.069. These findings indicated that it did not meet the standard value of the model fit. Accordingly, two pessimism (#2, #4) and two optimism (#1, #5) variables were removed based on the squared multiple correlation value. As a result, the fit of the modified model was described as follows: RMR = 0.055, NFI = 0.904, CFI = 0.923, IFI = 0.923, and RMSEA = 0.076, which are considered acceptable.

Next, three methods were used to verify convergent validity: standardized regression weights, construct reliability, and average variance extracted (AVE). The specific CFA results are listed in Table 2. The range of standardized regression coefficients for all the variables was 0.663–0.908. In addition, construct reliability (suitable when higher than 0.700) ranged from 0.854 to 0.946 and the AVE (suitable when higher than 0.500) ranged from 0.547 to 0.814. Thus, convergent validity was determined by satisfying all the three conditions.

Next, to verify discriminant validity, the correlations between the constructs and the AVE were compared. Table 3 shows the verification results of discriminant validity according to the correlation of each variable. The discriminant validity is verified by selecting the two variables with the highest correlation and comparing the squared value with the value of the mean variance ejection. The square of the correlation coefficient of “optimism 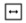 self-regulation ability”, which had the highest correlation, was 0.491, which was lower than the AVEs for optimism (0.547) and pessimism (0.588). Thus, discriminant validity between the variables was secured.

### 2.4. Descriptive Statistical Analysis

The overall results and those related to subfactors for sports participation, optimism/pessimism, self-regulation, and coronavirus-related stress are presented as descriptive statistics in Table 4. The mean values were distributed from 2.00 to 3.75, and the standard deviation was distributed from 0.77 to 1.22. In general, when skewness was <±3.0 and kurtosis was <±8.0 (the criteria for univariate normality violation), the conditions for normal distribution were fit [30,31]. Our analysis indicated that the absolute value of skewness was distributed in the range from 0.076 to 0.458, and that the absolute value of kurtosis was distributed in the range from 0.011 to 0.850. Thus, the normality of the structural equation was satisfied. In addition, Cronbach’s α, which verifies the degree of intra-item consistency, was used to verify the reliability of the scales used in the study. The analysis indicated that the scales had high internal consistency.

### 2.5. Data Collection and Analysis

Prior to this study, a pilot study involving 210 adolescents in the Jeollabuk-do Province was conducted from 21–25 June 2021. In the main study, data were collected from 626 people nationwide using the online questionnaire survey method through the Naver Survey Form presented online (www.naver.com) from 13–22 July 2021. The following data analyses were performed using SPSS 21.0 and AMOS 21.0 (IBM Corp., Armonk, NY, USA). According to the central limit theorem, if the number of participants is over 30, the data may be considered to be normally distributed and reliable, and parametric analyses may be used [32]. Thus, (1) frequency analysis was performed to confirm the general characteristics of the participants, (2) confirmatory factor analysis was performed to verify the validity of the research tool and the suitability of the research model, and (3) Cronbach’s α was used to verify the reliability. (4) Independent samples *t*-tests and one-way analyses of variance (ANOVAs) were performed to verify the differences in each variable according to the demographic characteristics (sex, school level, and frequency, intensity, and period of exercise). (5) Considering the errors observed for sports participation, optimism, pessimism, self-regulation, and coronavirus-related stress, multimedia analysis was performed via path analysis and bootstrapping for more accurate relationship verification. A phantom variable was used to verify the indirect effect of the multiparameter model through bootstrapping. The phantom variable is a nonexistent variable and is created by fixing the variance to 0 and fixing the path of the indirect effect to be measured to the factor loading value. Even if a phantom variable is added, all the parameters of the variable are fixed, and thus, there is no effect on the model fit [33]. We analyzed the 95% confidence interval and used 2000 bootstrap samples.

## 3. Results

### 3.1. Differences between Groups for Each Variable

#### 3.1.1. Differences in Each Variable According to Sex

Table 5 shows the results of each variable according to sex. Specifically, in all the subdomains of sports participation, scores of the male students were significantly higher than those of the female students. Scores for the pessimism variable were significantly lower among the male students than among the female students, while scores for the optimism variable were significantly higher among the male students than among the female students. For the self-regulation variable, the male students had significantly higher scores than the female students. In addition, scores for coronavirus-related stress were significantly lower among the male students than among the female students.

#### 3.1.2. Differences in Each Variable According to School Level

Differences in each variable according to school level are shown in Table 6. Our analysis indicated that there were no significant differences between the middle school students and the high school students for any variable except the compulsive component of coronavirus-related stress. Scores for the compulsive measure were significantly lower among the middle school students than among the high school students.

#### 3.1.3. Differences in Each Variable According to the Frequency of Physical Activity Participation Per Week

Differences in each variable according to the frequency of physical activity participation per week are presented in Table 7. Specifically, sports participation (affective participation (*F* = 68.112, *p* < 0.001), cognitive participation (*F* = 109.541, *p* < 0.001), behavioral participation (*F* = 165.913, *p* < 0.001)), pessimism (*F* = 26.387, *p* < 0.001), optimism (*F* = 45.189, *p* < 0.001), and self-regulation (reflecting (*F* = 32.475, *p* < 0.001), adjustment (F = 25.718, *p* < 0.001), monitoring (*F* = 17.379, *p* < 0.001), and planning (*F* = 22.596, *p* < 0.001)) differed significantly between the groups. There were also significant differences in the scores on the coronavirus-related stress variables including danger and contamination (*F* = 6.951, *p* < 0.001), xenophobia (*F* = 9.746, *p* < 0.001), and traumatic scores (*F* = 9.659, *p* < 0.001). However, there were no significant differences in the compulsiveness scores (*F* = 1.772, *p* = 0.151) between the groups.

#### 3.1.4. Differences in Each Variable According to the Amount of Time Spent Engaged in Physical Activity

Table 8 shows the differences in each variable according to the amount of time spent engaged in physical activity. Sports participation (affective participation (*F* = 86.838, *p* < 0.001), cognitive participation (*F* = 153.139, *p* < 0.001), behavioral participation (*F* = 210.369, *p* < 0.001)), pessimism (*F* = 33.997, *p* < 0.001), optimism (*F* = 60.777, *p* < 0.001), and self-regulation (reflecting (*F* = 31.355, *p* < 0.001), adjusting (*F* = 31.649, *p* < 0.001), monitoring (*F* = 16.692, *p* < 0.001), and planning (*F* = 15.977, *p* < 0.001)) scores differed based on the amount of time spent engaged in physical activity. In addition, there were significant differences in the scores for the coronavirus-related stress variables including danger and contamination (*F* = 11.360, *p* < 0.001), xenophobia (*F* = 15.567, *p* < 0.001), and traumatic scores (*F* = 14.986, *p* = 0.513). On the other hand, there were no significant differences in compulsiveness scores (*F* = 2.475, *p* = 0.061) based on the amount of time spent engaged in physical activity.

#### 3.1.5. Differences in Each Variable According to the Duration of Physical Activity Participation

Table 9 shows the differences in each variable according to the duration of physical activity participation. Sports participation (affective participation (*F* = 54.398, *p* < 0.001), cognitive participation (*F* = 99.020, *p* < 0.001), behavioral participation (*F* = 120.752, *p* < 0.001)), pessimism (*F* = 28.978, *p* < 0.001), optimism (*F* = 44.197, *p* < 0.001), and self-regulation (reflecting (*F* = 24.756, *p* < 0.001), adjusting (*F* = 28.494, *p* < 0.001), monitoring (*F* = 16.955, *p* < 0.001), planning (*F* = 16.709, *p* < 0.001)) scores differed significantly based on the duration of physical activity participation. In addition, scores for the coronavirus-related stress variables including danger and contamination (*F* = 8.404, *p* < 0.001), xenophobia (*F* = 9.463, *p* < 0.001), and traumatic scores (*F* = 7.018, *p* < 0.001) differed significantly according to the duration of physical activity participation. On the other hand, there were no significant differences in compulsiveness scores (*F* = 2.732, *p* = 0.430) based on the duration of physical activity participation.

### 3.2. Path Analysis

In this study, five latent variables (sports participation, optimism, pessimism, self-regulation, and coronavirus-related stress) were constructed to test the hypothetical model. The model also included 21 observational variables (three for sports participation, five for optimism, five for pessimism, four for self-regulation, and four for coronavirus-related stress). Before the path analysis, the fit of the set model was verified, and the fit of the hypothetical model was evaluated as acceptable, based on the following: RMR = 0.080, NFI = 0.904, IFI = 0.922, CFI = 0.921, and RMSEA = 0.079.

The results of the path analysis are shown in Table 10. First, the path coefficient for the effect of sports participation on optimism was 0.710 (*p* < 0.001), which was statistically significant, indicating that sports participation exerts a positive (+) effect on optimism. Second, the path coefficient for the effect of sports participation on coronavirus-related stress was −0.151 (*p* < 0.05), which was statistically significant, indicating that sports participation exerts a negative (–) effect on coronavirus-related stress. Third, the path coefficient for the effect of sports participation on pessimism was −0.541 (*p* < 0.001), which was statistically significant, indicating that sports participation exerts a negative (–) effect on pessimism. Fourth, the path coefficient for the effect of sports participation on self-regulation was 0.159 (*p* < 0.01), which was statistically significant, indicating that sports participation exerts a positive (+) effect on self-regulation. Fifth, the path coefficient for the effect of optimism on self-regulation was 0.523 (*p* < 0.001), which was statistically significant, indicating that optimism has a positive (+) effect on self-regulation. Sixth, the path coefficient for the effect of optimism on coronavirus-related stress was −0.211 (*p* < 0.001), which was statistically significant, indicating that optimism exerts a negative (–) effect on coronavirus-related stress. Seventh, the path coefficient for the effect of pessimism on self-regulation was −0.110 (*p* < 0.01), which was statistically significant, indicating that pessimism exerts a negative (–) effect on self-regulation. The path coefficient for the effect of pessimism on coronavirus-related stress was 0.567 (*p* < 0.001), which was statistically significant, indicating that pessimism exerts a positive (+) effect on coronavirus-related stress. Finally, the path coefficient for the effect of self-regulation on coronavirus-related stress was −0.103 (*p* < 0.05), which was statistically significant, indicating that self-regulation exerts a negative (–) effect on coronavirus-related stress.

#### Analysis of Mediating Effects

**Figure 1:**
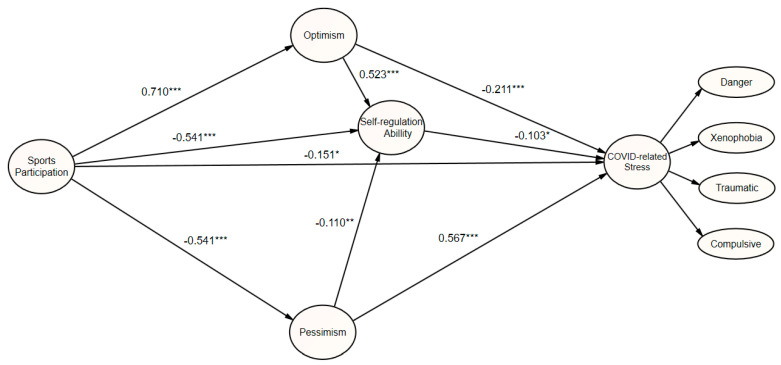
Structural model of mediating relationships; * *p* < 0.05; ** *p* < 0.01; *** *p* < 0.001.

The mediating effects identified through bootstrapping analysis of the relationship between sports participation and coronavirus-related stress are shown in Table 11 and Figure 1. Specifically, the singular mediating effect of optimism on the relationship between sports participation and coronavirus-related stress was 0.106 (*p* = 0.113), while the mediating effect of self-regulation was 0.023 (*p* = 0.050), indicating that there was no statistically significant mediating effect. However, the mediating effect of pessimism was −0.307 (*p* = 0.001), which was statistically significant. That is, in addition to its direct negative (–) effect on coronavirus-related stress, sports participation exerts an indirect effect on coronavirus-related stress through inertia control at a significance level of *p* < 0.01. Therefore, pessimism is an important mediating variable of coronavirus-related stress. In the analysis of multiple mediating effects, the mediating effect of the sports participation → pessimism → self-regulation → coronavirus-related stress pathway was 0.009 (*p* = 0.065), indicative of no mediating effect. However, the mediating effect of sports participation → optimism → self-regulation → mediating effect of coronavirus-related stress was 0.054 (*p* = 0.043), which was statistically significant.

## 4. Discussion

This study investigated the relationships between participation in sports, optimism/pessimism, self-regulation, and coronavirus-related stress among Korean adolescents in the context of the COVID-19 pandemic. First, there were significant differences in each variable according to demographic characteristics. Specifically, for all the sub-variables of sports participation, scores were significantly higher among the male students than among the female students. Scores for the pessimism variable were significantly lower among the male students than among the female students, while those for optimism were significantly higher among the male students than among the female students. Scores for self-regulation were significantly higher for the male students than for the female students, while those for coronavirus-related stress were significantly lower among the male students than among the female students. These results are consistent with those of Shevlin et al. [34] and Lee [29] who reported similar results for middle and high school students. However, given that Qi et al. [35] reported no sex differences in stress due to COVID-19, follow-up studies are required.

No significant differences in the variables related to sports participation, optimism, or self-regulation ability were observed according to school level. In terms of coronavirus-related stress, scores for the compulsive category were significantly lower among the middle school students than among the high school students. The finding that there is no difference in physical activity participation between middle school students and high school students is inconsistent with some previous studies [36,37]. The school curriculum significantly affects the physical activity of adolescents [36]; however, due to COVID-19, the students were not engaged in the physical activity typically performed at school (physical education time, sports club time). This may explain the similarity in the level of physical activity participation between the middle school students and the high school students, as similar findings have been reported for situations in which physical activity is restricted, such as prohibition of the use of sports facilities in schools [29].

In addition, higher levels of physical activity (frequency, intensity, and duration), were associated with higher scores for sports participation, optimism, and self-regulation and with lower scores for pessimism and coronavirus-related stress. These results are in line with those of Koo and Lee [38] who reported that participation in a physical activity-based recreation program had a positive effect on optimism in terms of frequency, intensity, and duration. In addition, according to the study by Qi et al. [35] which found a negative correlation between the level of physical activity and coronavirus-related stress, individuals who engaged in regular physical activity had higher levels of self-esteem, optimism, and happiness than those who were not physically active. Cekin [39] further highlighted this possibility, in support of the findings presented herein. In particular, Kybartas et al. [40] reported that moderate-intensity physical activity is not correlated with improvements in self-regulation, although correlations were observed for high-intensity physical activity. This is very similar to the results of this study, in which differences between each variable occurred only for high-intensity and long-term physical activity.

The findings presented herein indicate that sports participation had a positive effect on optimism and a negative effect on pessimism in Korean adolescents. Pavey et al. [41] observed a significant relationship between physical activity and optimism in young and middle-aged women, supporting the results reported herein. Thus, evidence suggests that physical activity plays an important role in creating a positive cognitive attitude toward the future [39] and that social and environmental changes should be initiated to promote physical activity among adolescents.

Sports participation had a statistically significant positive effect on self-regulation in Korean adolescents. This result is related to the finding that physical activity impacts the formation of a positive self-concept [22]. In addition, it is in line with the finding that the students who engaged in physical activity less frequently and were passive when engaged in physical activity were more prone to psychological problems such as depression and anxiety [42].

Sports participation exerted a statistically significant negative effect on coronavirus-related stress. Vogel et al. [43] reported that the participants who engaged in physical activity experienced lower levels of coronavirus-related stress than the inactive participants using stress-relieving strategies such as talking or playing with friends and family, exercising outdoors, and listening to music. This is consistent with the results of this study. These results are also consistent with a previous study on Chinese adolescents with similar environmental and physical conditions as those of Korean adolescents. As a result, physiological exercise helped alleviate negative emotions. Further, it has been shown that negative emotions may be alleviated more significantly with weekly physical activity of 2500 metabolic equivalents of energy [44].

Optimism exerted a positive effect on self-regulation, while pessimism exerted a negative effect on self-regulation. This result is in accordance with the previous finding [21] that optimistic individuals can engage in self-regulation for a longer period of time. Thus, when self-regulation ability is high, individuals can continue to perform tasks that require persistence for a longer duration. In addition, optimism plays an important role in sustaining and enhancing self-regulation [20,45,46,47], which is in agreement with the results reported herein. As such, optimistic people are more likely to engage in many goals simultaneously, and this characteristic increases their chances of achieving them in the long run. On the other hand, some studies have reported that optimistic individuals may struggle with conflicting goals in the short term [48]. Based on the results of previous studies, it can be inferred that adolescents with high optimism can handle stress well because of their high self-regulation ability. However, personally dealing with continuous stress during adolescence may lead to the accumulation of latent stress and cause greater conflict. Therefore, it is important to think about ways to increase the ability to overcome stress through appropriate physical activity rather than through simply avoiding stress.

The results reported herein also indicated that optimism exerts a negative effect on coronavirus-related stress, while pessimism exerts a positive effect on coronavirus-related stress, consistent with previous findings [18,48]. These results are also consistent with those of Reed [49] who investigated the mediating role of optimism between stress and life satisfaction. This suggests that people with higher levels of coronavirus-related stress are more likely to view psychological problems with more pessimism and less optimism. This may explain how stress can lead to greater pessimism and how low optimism can result in greater psychological problems [50,51,52,53]. Additionally, the beneficial effects of high levels of optimism and low levels of pessimism suggest the need to think about ways to develop optimism as it can aid individuals in overcoming coronavirus-related stress and attenuate psychological symptoms. In this study, self-regulation exerted a negative effect on coronavirus-related stress. This result is consistent with the finding that self-regulation style has a negative effect on stress [54,55]. In other words, self-regulation that relies on strategies that are in harmony with the self can help to reduce coronavirus-related stress.

This study had some limitations. In this work, meaningful results were derived by setting the degree of sports participation, optimism and pessimism, and self-regulation as variables that can affect coronavirus-related stress. However, diverse variables can influence coronavirus-related stress, many of which may not have been included in this study. Therefore, a multidimensional analysis that includes additional variables with the potential to impact coronavirus-related stress is required. Second, only Korean adolescents were assessed in this study; therefore, it would not be appropriate to generalize the study results to other ethnicities. Future studies should perform these assessments in populations that reflect the various characteristics of different ethnicities.

## 5. Conclusions

The results of this study suggest that sports participation not only directly relieves coronavirus-related stress, but also influences the factors that affect this stress. These findings highlight the need for youth educational institutions to encourage adolescents to participate in sports and for organizing bodies to suggest various policies and provide education that can assist them in properly coping with and overcoming coronavirus-related stress by strengthening their optimistic attitude and self-regulation ability. Therefore, follow-up studies may be conducted to gain insight into the methods of promoting adolescent sports participation. This may be achieved by analyzing factors that affect participation in sports in the context of the COVID-19 pandemic. For example, internal factors, such as individual motivation, and external factors, such as the opening of school and public sports facilities, physical education classes, and sports programs (both indoor and outdoor) and family support, may be considered.

## Figures and Tables

**Table 1:** Demographic characteristics of participants.

Variables	Classification	Pilot Test		Main Study	
		*n*	%	*n*	%
Sex	Male	112	53%	300	48%
	Female	98	47%	326	52%
School level	Middle school	135	64%	425	68%
	High school	75	36%	201	32%
Frequency of physical activity participation per week	Never	86	41%	167	26%
	Once a week	49	23%	87	14%
	2–3 times a week	52	25%	173	28%
	4 times a week or more	23	11%	199	32%
Amount of time spent engaged in physical activity	None	87	41%	153	25%
	30 min or less	61	29%	118	19%
	31–60 min	37	18%	209	33%
	60 min or more	25	12%	146	23%
Duration of physical activity participation	No participation	86	41%	176	28%
	3 months or less	56	27%	159	26%
	3–6 months	43	20%	108	17%
	6 months or more	25	12%	183	29%
Total		210	100%	626	100%

**Table 2:** Results of the confirmatory factor analysis (CFA).

Variables		Nonstandardized Coefficient	Standard Error	Critical Ratio	*p*	Standardized Coefficient	Construct Reliability	Average Variance Extracted
Sports participation	Affective	1.000	–	–	–	0.766	0.877	0.705
	Cognitive	1.687	0.072	23.374	<0.001 ***	0.908		
	Behavioral	1.505	0.067	22.517	<0.001 ***	0.863		
Pessimism	P1	1.000	–	–		0.741	0.884	0.588
	P3	0.959	0.054	17.878	<0.001 ***	0.722		
	P5	1.029	0.052	19.887	<0.001 ***	0.797		
	P6	1.093	0.051	21.522	<0.001 ***	0.860		
	P7	1.049	0.05	20.973	<0.001 ***	0.838		
Optimism	O7	1.000	–	–		0.693	0.858	0.547
	O6	1.259	0.068	18.412	<0.001 ***	0.821		
	O4	1.309	0.079	16.534	<0.001 ***	0.727		
	O3	1.272	0.074	17.285	<0.001 ***	0.764		
	O2	1.137	0.071	16.113	<0.001 ***	0.707		
Self-regulation	Reflecting	1.000	–	–		0.898	0.946	0.814
	Adjusting	0.988	0.030	33.368	<0.001 ***	0.902		
	Monitoring	0.886	0.030	29.298	<0.001 ***	0.846		
	Planning	0.878	0.035	25.33	<0.001 ***	0.783		
Coronavirus-related stress	Danger and contamination	1.000	–	–		0.754	0.854	0.594
	Xenophobia	1.06	0.065	16.418	<0.001 ***	0.718		
	Traumatic	0.997	0.056	17.669	<0.001 ***	0.787		
	Compulsive	0.801	0.053	15.232	<0.001 ***	0.663		

*** *p* < 0.001, tested by confirmatory factor analysis.

**Table 3:** Verification of discriminant validity.

Variables	Correlation between the Constructs					Average Variance Extracted
	Sports Participation	Pessimism	Optimism	Self-Regulation	Coronavirus-Related Stress	
Sports participation	1.000	–	–	–	–	0.705
Pessimism	0.498 ***	1.000	–	–	–	0.588
Optimism	0.676 ***	−0.669 ***	1.000	–	–	0.547
Self-regulation	0.562 ***	−0.503 ***	0.701 ***	1.000	–	0.814
Coronavirus-related stress	−0.257 ***	0.497 ***	−0.214 ***	−0.117 *	1.000	0.594

* *p* < 0.05, *** *p* < 0.001, tested by correlation analysis.

**Table 4:** Descriptive statistics (five-point Likert scale).

Variables		Mean	Standard Deviation	Skewness	Kurtosis	Cronbach’s α
Sports participation	Affective participation	3.61	0.86	−0.123	−0.065	0.629
	Cognitive participation	2.93	1.22	0.244	−0.850	0.939
	Behavioral participation	3.03	1.15	0.215	−0.751	0.801
Pessimism		2.31	0.88	0.196	−0.365	0.892
Optimism		3.74	0.81	−0.092	−0.376	0.859
Self-regulation	Reflecting	3.65	0.86	−0.243	−0.097	0.844
	Adjusting	3.64	0.80	−0.262	−0.044	0.839
	Monitoring	3.54	0.77	−0.346	0.179	0.844
	Planning	3.45	0.82	−0.458	0.299	0.794
Coronavirus-related stress	Danger and contamination	2.97	0.89	−0.121	−0.154	0.848
	Xenophobia	2.92	0.99	0.096	−0.508	0.918
	Traumatic	2.00	0.85	0.639	0.011	0.868
	Compulsive	2.57	0.81	0.076	−0.178	0.725

**Table 5:** Differences in all the variables according to sex.

	Sort	Mean ± Standard Deviation		*t*-Value	*p*
Variables		Male (*n* = 300)	Female (*n* = 326)		
Affective participation		3.90 ± 0.91	3.34 ± 0.71	8.618	<0.001 ***
Cognitive participation		3.54 ± 1.24	2.36 ± 0.88	13.580	<0.001 ***
Behavioral participation		3.57 ± 1.17	2.54 ± 0.87	12.357	<0.001 ***
Pessimism		2.11 ± 0.85	3.57 ± 0.73	−5.518	<0.001 ***
Optimism		3.92 ± 0.82	3.52 ± 0.68	5.554	<0.001 ***
Reflecting		3.76 ± 0.88	3.54 ± 0.74	3.390	0.001 **
Adjusting		3.74 ± 0.86	3.54 ± 0.73	3.082	0.002 **
Monitoring		3.62 ± 0.75	3.47 ± 0.78	2.546	0.110
Planning		3.51 ± 0.78	3.39 ± 0.85	1.778	0.076
Danger and contamination		2.76 ± 0.86	3.16 ± 0.88	−5.818	<0.001 ***
Xenophobia		2.73 ± 0.95	3.08 ± 1.00	−4.361	<0.001 ***
Traumatic		1.93 ± 0.92	2.07 ± 0.78	−2.090	0.037 *
Compulsive		2.43 ± 0.76	2.70 ± 0.84	−4.265	<0.001 ***

* *p* < 0.05, ** *p* < 0.01, *** *p* < 0.001, tested by independent samples *t*-tests.

**Table 6:** Differences in all the variables according to school level.

	Sort	Mean ± Standard Deviation		*t*-Value	*p*
Variables		Middle School Students (*n* = 425)	High School Students (*n* = 201)		
Affective participation		3.63 ± 0.85	3.57 ± 0.88	0.842	0.400
Cognitive participation		2.94 ± 1.23	2.89 ± 1.21	0.521	0.603
Behavioral participation		3.06 ± 1.15	2.97 ± 1.14	0.941	0.347
Pessimism		2.30 ± 0.91	2.33 ± 0.81	−0.285	0.776
Optimism		3.79 ± 0.81	3.65 ± 0.81	1.932	0.054
Reflecting		3.66 ± 0.85	3.61 ± 0.75	0.661	0.509
Adjusting		3.64 ± 0.83	3.63 ± 0.73	0.154	0.878
Monitoring		3.55 ± 0.78	3.52 ± 0.74	0.449	0.654
Planning		3.48 ± 0.82	3.39 ± 0.82	1.258	0.209
Danger and contamination		2.96 ± 0.90	2.98 ± 0.87	−0.334	0.739
Xenophobia		2.92 ± 1.00	2.90 ± 0.97	0.235	0.814
Traumatic		2.01 ± 0.87	1.99 ± 0.82	0.273	0.785
Compulsive		2.50 ± 0.81	2.73 ± 0.79	−3.342	0.001 **

** *p* < 0.01, tested by independent samples *t*-tests.

**Table 7:** Differences in all variables according to the frequency of physical activity participation per week.

	Sort	Mean ± Standard Deviation				*F*	*p*	Post Hoc
Variables		A (*n* = 167)	B (*n* = 87)	C (*n* = 173)	D (*n* = 199)			
Affective participation		3.18 ± 0.79	3.45 ± 0.62	3.39 ± 0.69	4.22 ± 0.81	68.112	<0.001 ***	A, B, C < D
Cognitive participation		2.21 ± 0.92	2.61 ± 0.89	2.60 ± 0.92	3.95 ± 1.14	109.541	<0.001 ***	A < B, C < D
Behavioral participation		2.18 ± 0.80	2.75 ± 0.82	2.77 ± 0.80	4.10 ± 0.96	165.913	<0.001 ***	A < B, C < D
Pessimism		2.49 ± 0.76	2.60 ± 0.82	2.48 ± 0.70	1.88 ± 0.97	26.387	<0.001 ***	A, B, C < D
Optimism		3.43 ± 0.72	3.58 ± 0.61	3.55 ± 0.72	4.24 ± 0.81	45.189	<0.001 ***	A, B, C < D
Reflecting		3.47 ± 0.77	3.32 ± 0.74	3.48 ± 0.70	4.08 ± 0.81	32.475	<0.001 ***	A, B, C < D
Adjusting		3.46 ± 0.73	3.32 ± 0.76	3.53 ± 0.71	4.02 ± 0.83	25.718	<0.001 ***	A, B, C < D
Monitoring		3.37 ± 0.79	3.31 ± 0.76	3.47 ± 0.72	3.84 ± 0.70	17.379	<0.001 ***	A, B, C < D
Planning		3.27 ± 0.89	3.15 ± 0.79	3.36 ± 0.75	3.81 ± 0.70	22.596	<0.001 ***	A, B, C < D
Danger and contamination		3.08 ± 0.92	3.09 ± 0.85	3.07 ± 0.84	2.73 ± 0.89	6.951	<0.001 ***	A, B, C < D
Xenophobia		2.99 ± 1.03	3.09 ± 0.93	3.10 ± 0.95	2.61 ± 0.96	9.746	<0.001 ***	A, B, C < D
Traumatic		2.06 ± 0.86	2.17 ± 0.78	2.16 ± 0.78	1.75 ± 0.88	9.659	<0.001 ***	A, B, C < D
Compulsive		2.61 ± 0.88	2.70 ± 0.82	2.58 ± 0.82	2.48 ± 0.73	1.772	0.151	–

*** *p* < 0.001, tested by one-way analyses of variance; A: none, B: once a week, C: 2–3 times a week, D: 4 times a week or more.

**Table 8:** Differences in all the variables according to the amount of time spent engaged in physical activity.

	Sort	Mean ± Standard Deviation				*F*	*p*	Post Hoc
Variables		A (*n* = 153)	B (*n* = 118)	C (*n* = 209)	D (*n* = 146)			
Affective participation		3.14 ± 0.81	3.45 ± 0.67	3.47 ± 0.67	4.42 ± 0.74	86.838	<0.001 ***	A < C, D, B < D, C < D
Cognitive participation		2.21 ± 0.93	2.45 ± 0.91	2.75 ± 0.91	4.32 ± 0.97	153.139	<0.001 ***	A, B < C < D
Behavioral participation		2.16 ± 0.80	2.68 ± 0.82	2.91 ± 0.81	4.42 ± 0.82	210.369	<0.001 ***	A < C, D, B < D, C < D
Pessimism		2.50 ± 0.78	2.55 ± 0.79	2.45 ± 0.77	1.72 ± 0.93	33.997	<0.001 ***	A, B, C < D
Optimism		3.39 ± 0.73	3.56 ± 0.64	3.62 ± 0.71	4.42 ± 0.76	60.777	<0.001 ***	A < C, D, B < D, C < D
Reflecting		3.43 ± 0.78	3.46 ± 0.74	3.53 ± 0.74	4.18 ± 0.82	31.355	<0.001 ***	A, B, C < D
Adjusting		3.44 ± 0.75	3.44 ± 0.75	3.53 ± 0.74	4.16 ± 0.76	31.649	<0.001 ***	A, B, C < D
Monitoring		3.34 ± 0.79	3.35 ± 0.75	3.56 ± 0.75	3.88 ± 0.65	16.692	<0.001 ***	A, B, C < D
Planning		3.23 ± 0.87	3.27 ± 0.84	3.46 ± 0.75	3.81 ± 0.66	15.977	<0.001 ***	A, B, C < D
Danger and contamination		3.12 ± 0.91	3.04 ± 0.84	3.07 ± 0.91	2.60 ± 0.79	11.360	<0.001 ***	A, B, C < D
Xenophobia		3.00 ± 1.03	3.19 ± 0.91	3.02 ± 0.99	2.46 ± 0.87	15.567	<0.001 ***	A, B, C < D
Traumatic		2.02 ± 0.85	2.25 ± 0.78	2.17 ± 0.80	1.63 ± 0.87	14.986	<0.001 ***	A, B, C < D
Compulsive		2.62 ± 0.85	2.67 ± 0.84	2.58 ± 0.85	2.42 ± 0.67	2.475	0.061	–

*** *p* < 0.001, tested by one-way analyses of variance; A: none, B: 30 min or less, C: 31–60 min, D: 60 min or more.

**Table 9:** Differences in all the variables according to the duration of physical activity participation.

	Sort	Mean ± Standard Deviation				*F*	*p*	Post Hoc
Variables		A (*n* = 176)	B (*n* = 159)	C (*n* = 108)	D (*n* = 183)			
Affective participation		3.19 ± 0.78	3.45 ± 0.71	3.54 ± 0.71	4.19 ± 0.82	54.398	<0.001 ***	A < B, C < D
Cognitive participation		2.22 ± 0.93	2.56 ± 0.95	2.89 ± 0.95	3.96 ± 1.17	99.02	<0.001 ***	A < B, C < D
Behavioral participation		2.22 ± 0.80	2.82 ± 0.85	3.00 ± 0.90	4.03 ± 1.07	120.752	<0.001 ***	A < B, C < D
Pessimism		2.52 ± 0.78	2.52 ± 0.81	2.46 ± 0.71	1.84 ± 0.93	28.978	<0.001 ***	A, B, C < D
Optimism		3.44 ± 0.72	3.59 ± 0.69	3.57 ± 0.70	4.26 ± 0.81	44.197	<0.001 ***	A, B, C < D
Reflecting		3.44 ± 0.76	3.48 ± 0.77	3.53 ± 0.72	4.06 ± 0.81	24.756	<0.001 ***	A, B, C < D
Adjusting		3.44 ± 0.72	3.42 ± 0.77	3.54 ± 0.73	4.07 ± 0.78	28.494	<0.001 ***	A, B, C < D
Monitoring		3.35 ± 0.76	3.42 ± 0.80	3.47 ± 0.70	3.86 ± 0.69	16.955	<0.001 ***	A, B, C < D
Planning		3.26 ± 0.84	3.35 ± 0.83	3.31 ± 0.74	3.79 ± 0.74	16.709	<0.001 ***	A, B, C < D
Danger and contamination		3.11 ± 0.92	3.09 ± 0.85	2.99 ± 0.89	2.70 ± 0.85	8.404	<0.001 ***	A, B < D
Xenophobia		3.00 ± 1.03	3.11 ± 0.94	3.03 ± 0.97	2.60 ± 0.95	9.463	<0.001 ***	A, B, C < D
Traumatic		2.06 ± 0.86	2.18 ± 0.79	2.02 ± 0.68	1.78 ± 0.94	7.018	<0.001 ***	A, B < D
Compulsive		2.63 ± 0.87	2.67 ± 0.82	2.53 ± 0.76	2.44 ± 0.76	2.732	0.430	–

*** *p* < 0.001, tested by one-way analyses of variance; A: none, B: 3 months of less, C: 3–6 months, D: more than 6 months.

**Table 10:** Path coefficients of the structural model.

Hypothesis	Path			Standardized Regression Coefficient	Regression Coefficient	Standard Error	Critical Ratio	*p*
H1	Sports participation	→	Optimism	0.659	0.710	0.048	13.588	<0.001 ***
H2	Sports participation	→	Coronavirus-related stress	−0.156	−0.151	0.075	−2.081	0.037
H3	Sports participation	→	Pessimism	−0.656	−0.541	0.057	−11.541	<0.001 ***
H4	Sports participation	→	Self-regulation	0.173	0.159	0.066	2.641	0.008 **
H4	Optimism	→	Self-regulation	0.614	0.523	0.070	8.742	<0.001 ***
H5	Optimism	→	Coronavirus-related stress	−0.239	−0.211	0.054	−4.383	<0.001 ***
H6	Pessimism	→	Self-regulation	−0.098	−0.110	0.037	−2.641	0.008 **
H7	Pessimism	→	Coronavirus-related stress	0.483	0.567	0.049	9.808	<0.001 ***
H8	Self-regulation	→	Coronavirus-related stress	−0.098	−0.103	0.043	−2.259	0.024 *

* *p* < 0.05, ** *p* < 0.01, *** *p* < 0.001, tested by path analysis.

**Table 11:** Multiple mediating effects.

Path	Nonstandardized Coefficient	Standard Error	Standardized Coefficient	*p*
Direct effect				
Sports participation → coronavirus-related stress	−0.156	0.075	−0.151	0.037 *
Indirect effect				
Sports participation → optimism → coronavirus-related stress	0.109	0.071	0.106	0.113
Sports participation → pessimism → coronavirus-related stress	−0.317	0.042	−0.307	0.001 **
Sports participation → self-regulation → coronavirus-related stress	0.024	0.016	0.023	0.050
Sports participation → optimism → self-regulation → coronavirus-related stress	0.056	0.033	0.054	0.043 *
Sports participation → pessimism → self-regulation → coronavirus-related stress	0.009	0.008	0.009	0.065
Total indirect effect	−0.119	0.072	−0.115	0.101
Total effect	−0.275	0.048	−0.205	0.001 **

* *p* < 0.05, ** *p* < 0.01, tested by path analysis.

## Data Availability

The data presented in this study are available upon request to the authors. Some variables are restricted to preserve anonymity of the study participants.
